# Risk of sudden unexpected death in epilepsy (SUDEP) with lamotrigine and other sodium channel‐modulating antiseizure medications

**DOI:** 10.1002/epi4.12693

**Published:** 2023-02-15

**Authors:** Russell Nightscales, Sarah Barnard, Juliana Laze, Zhibin Chen, Gerard Tao, Clarissa Auvrez, Shobi Sivathamboo, Mark J. Cook, Patrick Kwan, Daniel Friedman, Samuel F. Berkovic, Wendyl D'Souza, Piero Perucca, Orrin Devinsky, Terence J. O'Brien

**Affiliations:** ^1^ Department of Neuroscience, Central Clinical School Monash University Melbourne Victoria Australia; ^2^ Department of Neurology Alfred Health Melbourne Victoria Australia; ^3^ Department of Neurology New York University Grossman School of Medicine New York New York USA; ^4^ Clinical Epidemiology, School of Public Health and Preventive Medicine Monash University Melbourne Victoria Australia; ^5^ Department of Medicine (The Royal Melbourne Hospital) The University of Melbourne Melbourne Victoria Australia; ^6^ Department of Neurology The Royal Melbourne Hospital Melbourne Victoria Australia; ^7^ NorthWestern Mental Health Melbourne Health Melbourne Victoria Australia; ^8^ Department of Medicine St. Vincent's Hospital, The University of Melbourne Fitzroy Victoria Australia; ^9^ Department of Medicine, Austin Health The University of Melbourne Heidelberg Victoria Australia; ^10^ Comprehensive Epilepsy Program, Department of Neurology Austin Health Heidelberg Victoria Australia

## Abstract

**Objective:**

In vitro data prompted U.S Food and Drug Administration warnings that lamotrigine, a common sodium channel modulating anti‐seizure medication (NaM‐ASM), could increase the risk of sudden death in patients with structural or ischaemic cardiac disease, however, its implications for Sudden Unexpected Death in Epilepsy (SUDEP) are unclear.

**Methods:**

This retrospective, nested case–control study identified 101 sudden unexpected death in epilepsy (SUDEP) cases and 199 living epilepsy controls from Epilepsy Monitoring Units (EMUs) in Australia and the USA. Differences in proportions of lamotrigine and NaM‐ASM use were compared between cases and controls at the time of admission, and survival analyses from the time of admission up to 16 years were conducted. Multivariable logistic regression and survival analyses compared each ASM subgroup adjusting for SUDEP risk factors.

**Results:**

Proportions of cases and controls prescribed lamotrigine (*P* = 0.166), one NaM‐ASM (*P* = 0.80), or ≥2NaM‐ASMs (*P* = 0.447) at EMU admission were not significantly different. Patients taking lamotrigine (adjusted hazard ratio [aHR] = 0.56; *P* = 0.054), one NaM‐ASM (aHR = 0.8; *P* = 0.588) or ≥2 NaM‐ASMs (aHR = 0.49; *P* = 0.139) at EMU admission were not at increased SUDEP risk up to 16 years following admission. Active tonic–clonic seizures at EMU admission associated with >2‐fold SUDEP risk, irrespective of lamotrigine (aHR = 2.24; *P* = 0.031) or NaM‐ASM use (aHR = 2.25; *P* = 0.029). Sensitivity analyses accounting for incomplete ASM data at follow‐up suggest undetected changes to ASM use are unlikely to alter our results.

**Significance:**

This study provides additional evidence that lamotrigine and other NaM‐ASMs are unlikely to be associated with an increased long‐term risk of SUDEP, up to 16 years post‐EMU admission.

**Key Points**

- We investigated if lamotrigine and sodium channel modulating anti‐seizure medications (NaM‐ASMs) are associated with an increased risk of Sudden Unexpected Death in Epilepsy (SUDEP).
- We identified 101 SUDEP cases and 199 matched living controls admitted for epilepsy video‐EEG monitoring across four tertiary centers in Australia & USA over an 18‐year period.
- Proportions of SUDEP cases and living controls prescribed NaM‐ASMs at the time of admission were similar.
- Patients prescribed NaM‐ASMs were not at an increased risk of future SUDEP and showed similar cumulative survival up to 16 years, compared to those who were not.
- Our study provides additional evidence that NaM‐ASMs are unlikely to be associated with an increased long‐term risk of SUDEP up to 16 years.

Lamotrigine is a commonly prescribed sodium channel‐modulating antiseizure medication (NaM‐ASM). In vitro data demonstrating lamotrigine activity against cardiac sodium channels and class IB antiarrhythmic action prompted U.S Food and Drug Administration (FDA) warnings that clinically relevant concentrations of lamotrigine could induce serious cardiac arrhythmias and increase the risk of sudden death in patients with structural or ischaemic cardiac disease.^1^ Class‐wide investigations of adverse cardiac event risk in NaM‐ASM use have since been recommended.^2^ Though the pathophysiological understanding of sudden unexpected death in epilepsy (SUDEP) is incomplete, postictal cardiac dysfunction and arrhythmia have been observed^3^, ^4^ and the implications of these warnings on SUDEP are unclear.

Following the FDA's warning, a rapid systematic review of lamotrigine and sudden death was conducted concluding there is currently insufficient evidence to support or refute the FDA's hypothesis.^5^ Reassuringly, a recent European population‐based study found no association between lamotrigine and new onset cardiac conduction abnormalities or all‐cause mortality in those with pre‐existing cardiac conditions, however modest increases in mortality from “epilepsy” and “unknown causes” were observed in lamotrigine users.^6^ Previously, an analysis from pooled data from four published case–control studies of SUDEP with live controls reported an association of lamotrigine use with an increased risk of SUDEP,^7^ but this has not been supported by analyses from clinical trials or other studies that controlled for the frequency of tonic–clonic seizures (TCS).^8^, ^9^, ^10^ There also remains concerned for the potential of synergism in NaM‐ASM polytherapy in SUDEP,^11^ though previous studies have reported that ASM regimes that include lamotrigine may reduce SUDEP risk independent of TCS frequency.^12^

The ongoing paucity of conclusive, clinical data on the risk of sudden death and use of NaM‐ASMs may deter clinicians from prescription with the potential to impact seizure control, quality of life, and SUDEP risk in people with epilepsy. Further studies in clinical populations examining the association of sodium channel‐modulating ASMs use and SUDEP risk, controlling for known SUDEP risk factors such as TCS frequency^7^, ^13^ are needed.

This study aims to investigate differences in lamotrigine and NaM‐ASM prescription between SUDEP cases and living epilepsy controls in cohorts of patients admitted to an Epilepsy Monitoring Unit (EMU), and their relative survival following admission. We hypothesized that if lamotrigine and other NaM‐ASMs are associated with increased SUDEP risk, we would see excess usage at the time of admission among SUDEP cases when compared to controls, and shorter survival after EMU admission in SUDEP cases in patients taking these drugs.

## 1 METHODS

### 1.1 Participants and study design

This international, multicentre, nested, case–control study retrospectively reviewed databases containing the records of 11 050 patients admitted to one of four epilepsy monitoring units (EMUs). Between 1995 and 2013, 4874 patients were admitted to three tertiary hospital EMUs in Melbourne, Australia: The Royal Melbourne Hospital (n = 2024), St Vincent's Hospital (n = 1091), and The Austin Hospital (n = 1759). Between 2008 and 2013, 6176 patients were admitted to NYU Langone Medical Centre, New York, USA. Patients in the American (US) cohort were included if they had at least one electroclinical seizure captured on video‐EEG. Patients in the Australian cohort were included if the patient met the diagnostic criteria for epilepsy. Epilepsy diagnosis and syndrome were determined in accordance with International League Against Epilepsy (ILAE) guidelines,^14^ by epileptologists and a multidisciplinary team (radiologists, neuropsychologists, neurophysiology scientists, and nursing staff), using video, EEG, neuropsychology, historical, and imaging (magnetic resonance imaging, positron emission tomography, single photon emission computed tomography) data. Of the 2807 Australian cases diagnosed with epilepsy, 2106 (75%) had at least one captured electroclinical seizure on video‐EEG. In total, 4980 patients were included in the study.

### 1.2 Identifying deaths and SUDEP cases

Patients included in the study were linked to national death registries to determine their living status. In the US cohort, patients who had records of a hospital or clinical visit in the six months prior to linkage were considered as living, and subsequently not linked to death registries. The final dates of follow‐up were October 1, 2018 and April 5, 2021 for Australian and US patients, respectively. Patients identified as deceased in both cohorts were considered potential SUDEP cases and underwent review. Deaths >16 years from admission were excluded. Circumstantial information was obtained for all potential SUDEP cases from a combination of coronial, autopsy, police, and post‐mortem toxicology reports, death certificates, and direct family or treating‐clinician correspondence. Two neurologists independently reviewed this information in each cohort (T.O'B and P.P—Australian cases, O.D and D.F—US cases), and classified deaths as SUDEP or non‐SUDEP, according to Nashef et al. criteria.^15^

Of the decedents, 102 SUDEP cases were identified (definite [n = 48], probable [n = 23], possible [n = 27], and near [n = 4]). SUDEP cases were matched with two living epilepsy controls for EMU admission site, age (±3 years), sex, and year of EMU admission (±1 year), with ≥5 years follow‐up. One unmatched SUDEP case was excluded. The final cohort included 101 SUDEP cases and 199 controls.

### 1.3 Clinical data

All data were sourced from the EMU databases, patients' hospital medical records, and death records. Demographic data included sex, age at EMU admission, and age at death or follow‐up. Clinical data included information on the age of epilepsy onset, epilepsy syndrome, etiology, a history of TCSs, nocturnal seizures or nocturnal TCS, and ASM non‐adherence in the 12 months preceding the EMU admission. Data collected from EMU admission included the number, type, and lateralization of seizures captured during admission, ASM use and total daily dosages, and medical, psychiatric, and neurosurgical history at the time of admission. A history of any cardiac disease was defined as documented congenital or acquired structural heart disease or cardiomyopathy, arrhythmia, or coronary artery disease. Electrocardiograms (ECGs) were not reviewed and specific arrhythmia types were not recorded. Seizure types were classified according to current ILAE guidelines.^16^ Follow‐up medications were obtained from post‐mortem toxicology, police reports, and medical records, with data closest to the time of death prioritized. In recognition that medications prescribed at death may not be detected by post‐mortem toxicological analyses, medications prescribed within 12 months of death as written in the medical record were prioritized over toxicological investigations, where relevant. ASM data within 12 months of death was available for 81 SUDEP cases.

### 1.4 Statistical analysis

The association of the type ASM use at the time of EMU admission with long‐term SUDEP risk was analyzed in two ways: (a) lamotrigine vs other ASMs; and (b) those on 1 NaM‐ASM or ≥2 NaM‐ASMs vs no NaM‐ASMs. NaM‐ASMs were defined as: carbamazepine, cenobamate, eslicarbazepine acetate, fosphenytoin, lacosamide, lamotrigine, oxcarbazepine, phenytoin, rufinamide, and zonisamide. Fisher's exact test analyzed categorical data and Mann–Whitney test continuous data.

Differences in ASM use between cases and controls at EMU admission were analyzed using multivariable logistic regression for each ASM group. Purposeful variable selection approach^17^ was used to control for potential confounding factors with univariable *P*‐value <0.2 in multivariable analyses. Age variables were centered at the median. Univariable Cox regression with the Wald test was used in survival analysis to identify independent SUDEP risk factors. Multivariable Cox regression compared differences in time‐to‐SUDEP between ASM groups.

To evaluate the effects of unknown ASM prescription at the time of follow‐up (i.e., possible lamotrigine misclassification), we performed Monte‐Carlo sensitivity analysis on non‐lamotrigine uses (defined as the proportion of patients who were on other ASMs at the time of EMU admission truly not exposed to lamotrigine in the subsequent study period) in cases and controls. Non‐lamotrigine sensitivity was set in a range of 0.1 to 0.9, incrementing by 0.1, and 1000 iterations were performed in each scenario. Further subgroup analysis used cases with available ASM information within 12 months of death was also performed.

A significance level of *P* < 0.05 was used for all analyses unless otherwise stated. All statistical analyses were performed using *Stata*, version 16 (StataCorp, College Station, Texas, USA).

### 1.5 Standard protocol approvals, registrations, and patient consents

Australian Human Research Ethics Committee approval for the study was provided by St Vincent's Hospital, Melbourne Health (The Royal Melbourne Hospital), and Austin Health (The Austin Hospital). Approval for data linkage to the Australian National Death Index was granted by the Australian Institute of Health and Welfare (AIHW) Ethics committee. Access to the Australian National Coronial Information System was granted by the Justice Human Research Ethics Committee. Approval for the inclusion of US cases was granted by the NYU Institutional Review Board.

## 2 RESULTS

### 2.1 Demographics and epilepsy characteristics

Demographics and epilepsy characteristics are summarized in Table 1. The median duration of follow‐up to determine deceased status following EMU admission was 9.82 years (interquartile range [IQR]: 5.52–14.1).

**TABLE 1:** Demographic and epilepsy characteristics in SUDEP cases and controls at EMU

		Controls at EMU (199)				Future SUDEP cases at EMU (101)				Future SUDEP cases at EMU, with ASM data within 12 months of death (81)			
		n	%	*Mean* (*St Dev*)	*Median* (*IQR*)	n	*%*	*Mean* (*St Dev*)	*Median* (*IQR*)	n	*%*	*Mean* (*St Dev*)	*Median* (*IQR*)
Follow‐up Duration		‐	‐	‐	*12.7* (*9.08–15.3*)	‐	‐	‐	*4.19* (*2.02–6.83*)	‐	‐	‐	*3.59* (*1.34–5.88*)
Basic demographics	Female	95	47.7%	‐	‐	48	47.5%	‐	‐	40	49%	‐	‐
	Male	104	52.3%	‐	‐	53	52.5%	‐	‐	41	51%	‐	‐
	Age at EMU admission (years)	‐	‐	33.4 (13.6)	‐	‐	‐	32.9 (12.8)	‐	‐	‐	33.3 (13.3)	‐
Site	Austin	57	28.6%	‐	‐	29	28.7%	‐	‐	24	30%	‐	‐
	RMH	53	26.6%	‐	‐	27	26.7%	‐	‐	21	26%	‐	‐
	St Vincent's	49	24.6%	‐	‐	25	24.8%	‐	‐	16	20%	‐	‐
	NYU	40	20.1%	‐	‐	20	19.8%	‐	‐	20	25%	‐	‐
Epilepsy etiology	Genetic/Presumed Genetic	31	15.6%	‐	‐	17	16.8%	‐	‐	12	15%	‐	‐
	Structural/Metabolic	78	39.2%	‐	‐	39	38.6%	‐	‐	30	37%	‐	‐
	Inflammatory/Infectious	4	2.0%	‐	‐	0	0.0%	‐	‐	0	0%	‐	‐
	Unknown	86	43.2%	‐	‐	45	44.6%	‐	‐	39	48%	‐	‐
Epilepsy classification	Focal	149	74.9%	‐	‐	68	67.3%	‐	‐	57	70%	‐	‐
	Generalized	24	12.1%	‐	‐	26	25.7%	‐	‐	13	16%	‐	‐
	Multifocal	15	7.5%	‐	‐	11	10.9%	‐	‐	8	9.9%	‐	‐
	Combined Focal & Generalized	6	3.0%	‐	‐	4	4.0%	‐	‐	2	2.5%	‐	‐
	Unknown/ND	5	2.5%	‐	‐	2	2.0%	‐	‐	1	1.2%	‐	‐

### 2.2 ASM prescribing and follow‐up

ASM prescribing at the EMU admission, and within 12 months of death for the SUDEP cases is summarized in Table 2. There were similar proportions of SUDEP cases and controls prescribed lamotrigine, one NaM‐ASM, or ≥2 NaM‐ASMs at EMU admission. The proportions of SUDEP cases prescribed lamotrigine, one NaM‐ASM or ≥2 NaM‐ASMs at EMU admission were comparable to those within 12 months of death. Many cases were on the same drug within 12 months of death as at EMU admission (72.7% remained on lamotrigine, 89.6% on one NaM‐ASM, and 47.3% on ≥2 NaM‐ASMs). Of those taking ≥2 NaM‐ASMs at the time of EMU admission, 93.8% remained on at least one NaM‐ASM within 12 months of death. Valproate, topiramate, and levetiracetam were the most prescribed non‐NaM‐ASMs at the time of admission, with similar overall rates of prescription at EMU and within 12 months of death (Table 2). Post‐mortem toxicology available in 14 patients prescribed lamotrigine within 12 months of death revealed subtherapeutic levels in 57.1% of cases and therapeutic levels in 42.9% of cases.

**TABLE 2:** Proportion of patients taking lamotrigine or not, and proportion of patients taking zero, one or two or more sodium channel modulators at the time of EMU admission and around the time of death

		Controls at EMU (199)				Future SUDEP cases at EMU (101)				SUDEP cases with ASM data within 12 months of death (81)			
		n	*%*	*Mean* (*St Dev*)	*Median* (*IQR*)	n	*%*	*Mean* (*St Dev*)	*Median* (*IQR*)	n	*%*	*Mean* (*St Dev*)	*Median* (*IQR*)
Basic demographics	Age at death (years)	‐	‐	n/a	‐	‐	‐	38.7 (14.0)	‐	‐	‐	38.1 (13.6)	‐
Lamotrigine only	Lamotrigine	78	39.2%	‐	‐	33	32.7%	‐	‐	27	33%	‐	‐
	No Lamotrigine	121	60.8%	‐	‐	68	67.3%	‐	‐	54	67%	‐	‐
NaM‐ASMs	0 NaM‐ASM	43	21.6%	‐	‐	21	20.8%	‐	‐	14	17%	‐	‐
	1 NaM‐ASM	111	55.8%	‐	‐	62	61.4%	‐		48	59%	‐	‐
	≥ 2 NaM‐ASMs	45	22.6%	‐	‐	18	17.8%	‐	‐	19	23%	‐	‐
Common non‐NaM‐ASMs	Valproate	73	36.7%	‐	‐	35	34.7%	‐	‐	28	35%	‐	‐
	Topiramate	44	22.1%	‐	‐	26	25.7%	‐	‐	22	27%	‐	‐
	Levetiracetam	47	23.6%	‐	‐	20	19.8%	‐	‐	21	26%	‐	‐

Abbreviations: EMU = EEG monitoring unit; IQR = Interquartile range; NaM‐ASM = sodium channel modulating anti‐seizure medication; ND = non‐diagnostic; SUDEP = sudden unexpected death in epilepsy; St Dev = Standard deviation.

After adjusting for epilepsy etiology, history of focal to bilateral tonic–clonic seizures (FBTCS), tonic or atonic seizures, autism spectrum disorder, developmental delay, and age of epilepsy onset, there was no significant difference in the proportion of SUDEP cases to controls prescribed lamotrigine at the time of EMU admission (adjusted odds ratio [aOR] = 0.66; 95% Confidence Interval [CI]: 0.36–1.19, *P* = 0.166), or those prescribed no NaM‐ASMs vs one (aOR = 0.91; 95% CI: 0.42–1.93, *P* = 0.80), no NaM‐ASMs vs ≥2NaM‐ASMs (aOR = 0.67; 95% CI: 0.25–1.90, *P* = 0.447), or one NaM‐ASM vs ≥2NaM‐ASMs (aOR = 1.33; 95% CI: 0.65–2.70, *P* = 0.44).

### 2.3 Survival analyses

**FIGURE 1:**
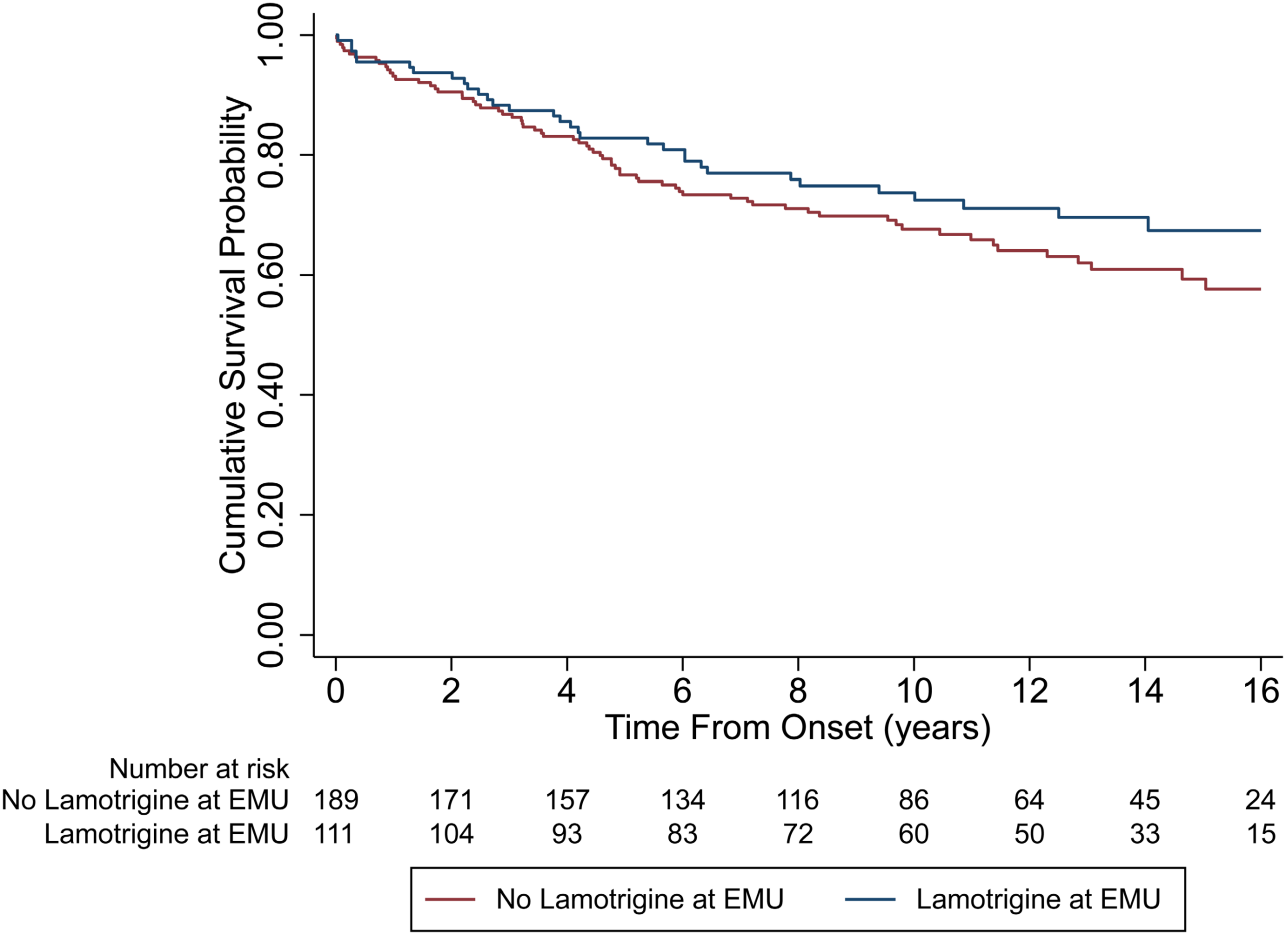
Univariable Kaplan Meyer curve comparing the probability of SUDEP between patients taking lamotrigine at the time of EMU admission to those not taking lamotrigine.

**FIGURE 2:**
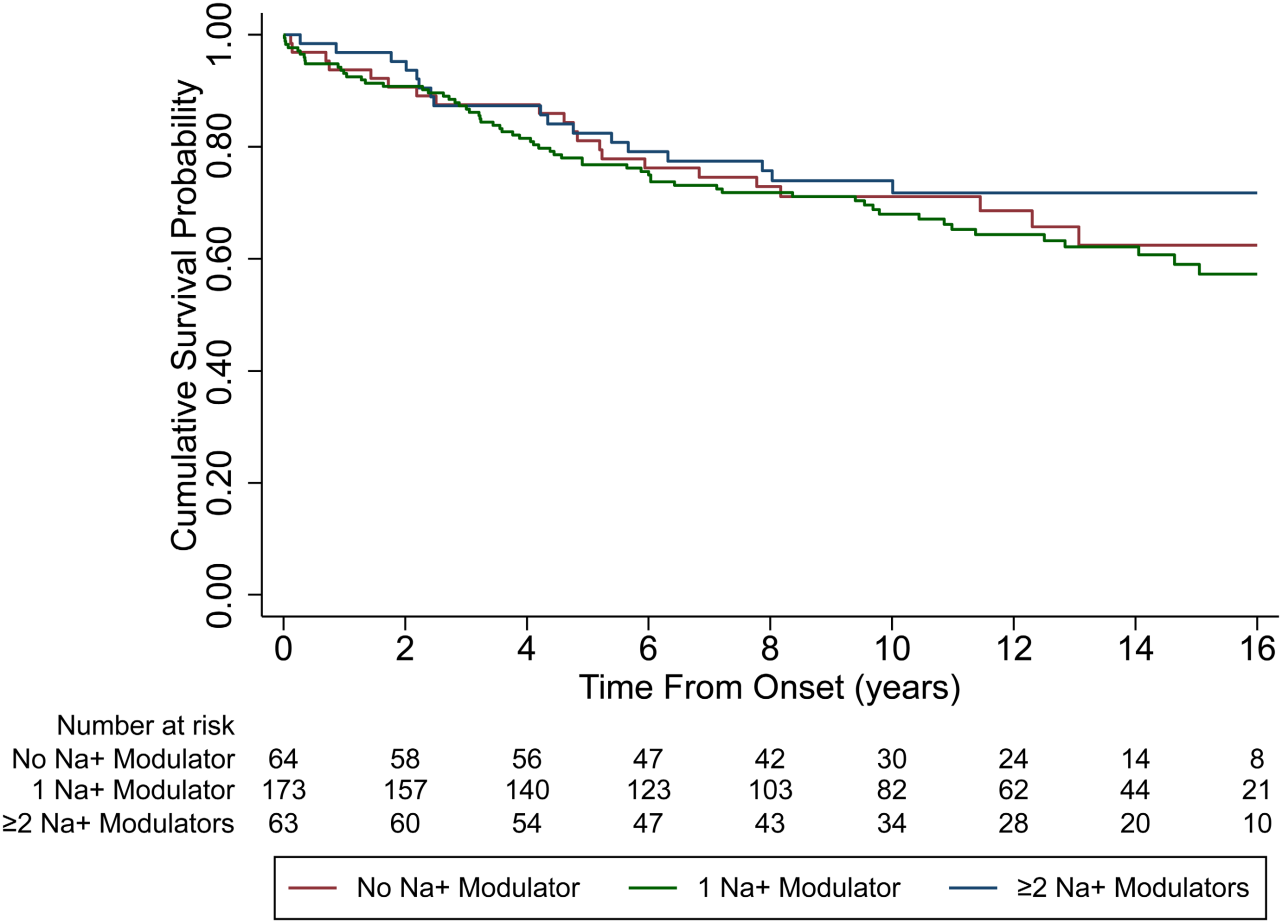
Univariable Kaplan Meyer curve comparing the probability of SUDEP between patients taking one, or two or more sodium channel blockers to those not taking any sodium channel blocker at the time of EMU admission.

The majority (87%) of patients had <16 years of follow‐up. Only one SUDEP case occurred after 16 years, at 21.2 years, which is considered an outlier as it is far above the upper fence (Q3 + 1.5*IQR) of time‐to‐event in SUDEP cases (median: 4.19; IQR: 2.02–6.83). Thus, the analysis was restricted to a maximum of 16 years to avoid an outlier effect. Univariable Kaplan–Meier curves show similar rates of cumulative survival probability from the time of EMU admission to 16 years of follow‐up between those taking lamotrigine at the time of admission and those who were not, and those taking one NaM‐ASM, or ≥2NaM‐ASMs compared those who were not (Figures 1 and 2, respectively).

### 2.4 Multivariable analysis of SUDEP risk

After controlling for univariable factors associated with SUDEP (*P* < 0.2) and removal of factors with only one patient (Table 3), multivariable cox regression analysis revealed no significant difference in SUDEP risk in those prescribed lamotrigine at the time of EMU admission compared to those who were not (adjusted hazard ratio [aHR] = 0.56; 95% CI: 0.31–1.01, *P* = 0.054). There was also no significant difference in SUDEP risk in those prescribed one NaM‐ASM or ≥2NaM‐ASMs at admission to those prescribed no NaM‐ASMs (aHR = 0.82; 95% CI: 0.40–1.68, *P* = 0.588 and aHR = 0.49; 95% CI: 0.19–1.26, *P* = 0.139, respectively). When including only definite or probable SUDEP cases and their matched controls, results were similar for those prescribed lamotrigine compared to those who were not, (aHR = 0.58; 95% CI: 0.29–1.17; *P* = 0.13) and those prescribed one NaM‐ASM (aHR = 1.27; 95% CI: 0.52–3.12; *P* = 0.60) or ≥2 NaM‐ASMs (aHR = 0.46; 95% CI: 0.15–1.63; *P* = 0.25) compared to those taking no NaM‐ASMs (Tables 4 and 5).

**TABLE 3:** Univariable analysis of factors associated with SUDEP at p < 0.2 and controlled for in the multivariable analyses

Factor	n	HR	*P* value
History of FAS	294	0.63	0.070
History of GTCS	295	1.82	***0.023***
History of Myoclonic seizures	295	0.27	0.062
History of Myoclonic Absence seizures^a^	295	26.2	***0.002***
History of Nocturnal seizures	277		0.064
Other seizures vs No nocturnal seizures		0.54	0.193
TCS vs No nocturnal seizures		1.47	0.095
TCS vs Other seizures		2.70	***0.039***
History of Anxiety	283	0.39	***0.042***
History of COPD^a^	283	39.8	**<*0.001***
Any TCS in 12 months preceding admission	221	3.25	**<*0.001***
Any nocturnal TCS in 12 months preceding admission	226	1.95	***0.037***
Median centered age of epilepsy onset	266	0.99	0.175

Abbreviations: COPD = chronic obstructive pulmonary disease; FAS = focal aware seizures; GTCS = generalized tonic–clonic seizures; HR = hazard ratio; TCS = tonic–clonic seizures.

^a^ Removed from multivariate analyses due to single patient events. Bolded *p* values = significant to *p* < 0.05.

**TABLE 4:** Multivariable survival analysis of factors associated with SUDEP in definite and probable SUDEP cases prescribed lamotrigine

Factor	aHR	95% CI	*P*‐value
Lamotrigine use	0.58	0.29–1.17	0.13
History of FAS	0.89	0.43–1.83	0.75
History of BTCS	2.15	0.93–4.98	0.074
History of myoclonic seizures	<0.001	N/A	1.00
History of Nocturnal seizures			
TCS vs No nocturnal seizures	1.48	0.73–2.88	0.29
Other seizures vs No nocturnal seizures	0.52	0.12–2.27	0.38
TCS vs Other seizures	2.79	0.60–12.9	0.19
History of anxiety	<0.001	N/A	1.00
History of structural heart disease	0.94	0.12–7.29	0.96
Any TCS in 12 months preceding admission	1.86	0.80–4.29	0.15
Median centered age of epilepsy onset	0.99	0.96–1.02	0.61

**TABLE 5:** Multivariable survival analysis of factors associated with SUDEP in definite and probable SUDEP cases prescribed one, two or more NaM‐ASMs

Factor	aHR	95% CI	*P*‐value
NaM‐ASM			
One NaM‐ASM vs No NaM‐ASM	1.27	0.52–3.12	0.60
≥2 NaM‐ASM vs No NaM‐ASM	0.49	0.15–1.63	0.25
≥2 NaM‐ASM vs One NaM‐ASM	2.59	0.97–6.90	0.058
History of FAS	0.86	0.41–1.80	0.69
History of BTCS	2.23	0.97–5.15	0.060
History of myoclonic seizures	<0.001	N/A	1.00
History of Nocturnal seizures			
TCS vs No nocturnal seizures	1.27	0.63–2.59	0.51
Other seizures vs No nocturnal seizures	0.53	0.12–2.34	0.41
TCS vs Other seizures	2.38	0.52–10.9	0.26
History of anxiety	<0.001	N/A	1.00
History of structural heart disease	0.98	0.13–7.47	0.98
Any TCS in 12 months preceding admission	2.10	0.91–4.84	0.082
Median centered age of epilepsy onset	0.99	0.96–1.02	0.62

Abbreviations: aHR = adjusted hazard ratio; CI = Confidence Interval; FAS = focal aware seizures; NaM‐ASM = sodium channel modulating anti‐seizure medication; N/A, not applicable owing to rare event number; TCS = tonic–clonic seizures.

Patients with a history of tonic–clonic seizures in the 12 months leading up to admission had a >2 times increased SUDEP risk than those without, irrespective of lamotrigine or NaM‐ASM use (aHR = 2.24; 95% CI: 1.07–4.68, *P* = 0.031 and aHR = 2.25; 95% CI: 1.09–4.67, *P* = 0.029, respectively). A history of structural heart disease was not associated with altered SUDEP risk in either analysis (aHR = 0.46; 95% CI: 0.06–3.62, *P* = 0.46 and aHR = 0.51, 95% CI: 0.07–3.99, *P* = 0.52, respectively).

### 2.5 Sensitivity analyses

Results from the Monte‐Carlo sensitivity analysis are presented in Table 6. While holding ASM use in SUDEP cases constant, as non‐lamotrigine sensitivity decreases in controls (i.e., more controls actually exposed to lamotrigine), lamotrigine starts to show an increasingly protective effect against SUDEP. On the other hand, lamotrigine would become associated with SUDEP if non‐lamotrigine sensitivity is lower than 70% in SUDEP cases while holding ASM use in controls constant. Among the 101 SUDEP cases, 81 had available ASM information within 12 months of death and 65 remained on the same ASM regimen as at the time of EMU admission. Of the 20 SUDEP cases who did not have available ASM information within 12 months of death, 6 were on lamotrigine at the time of EMU admission and 14 were not. This provides a minimum 68% non‐lamotrigine sensitivity in the SUDEP cases. Therefore, unless in the very rare case where all the non‐lamotrigine users were misclassified in the cases but none in the controls, the misclassification effect would not lead to contradictory results. Subgroup analyses confined to cases with available ASM information within 12 months of death and their matched controls also show similar results (Table 7).

**TABLE 6:** Monte‐Carlo sensitivity analysis of non‐lamotrigine users in the control and SUDEP groups

Group	Non‐LTG sensitivity	Average HR	Min HR	Max HR	Average OR	Min OR	Max OR
Controls	0.9	0.48	0.39	0.55	0.51	0.44	0.57
	0.8	0.40	0.32	0.48	0.39	0.33	0.46
	0.7	0.34	0.27	0.45	0.30	0.25	0.37
	0.6	0.29	0.22	0.38	0.23	0.17	0.30
	0.5	0.24	0.19	0.30	0.17	0.12	0.22
	0.4	0.20	0.14	0.25	0.12	0.08	0.16
	0.3	0.16	0.11	0.21	0.08	0.04	0.11
	0.2	0.12	0.09	0.16	0.05	0.03	0.07
	0.1	0.09	0.07	0.11	0.02	0.01	0.03
SUDEP cases	0.9	0.73	0.58	0.87	0.92	0.70	1.05
	0.8	0.93	0.72	1.18	1.26	0.96	1.50
	0.7	1.13	0.81	1.47	1.63	1.24	2.07
	0.6	1.43	1.05	1.88	2.22	1.67	2.88
	0.5	1.83	1.33	2.59	3.04	2.25	4.21
	0.4	2.42	1.68	3.93	4.30	3.17	6.32
	0.3	3.45	2.23	6.04	6.43	4.59	10.10
	0.2	5.17	3.22	10.22	10.02	7.34	16.98
	0.1	11.18	6.69	51.61	22.69	16.22	129.18

Abbreviations: HR = Hazard Ratio; LTG = Lamotrigine; OR = Odds Ratio; SUDEP = Sudden unexpected death in epilepsy.

**TABLE 7:** Subgroup sensitivity analyses of SUDEP cases with ASM information within 12 months of death, compared to controls using a) lamotrigine and b) any NaM‐ASM

	aHR	95% CI	*P*‐value
a) Lamotrigine	0.63	0.35–1.10	0.11
Active TCS	4.00	2.09–7.64	<0.001
Any cardiac history	2.07	0.63–6.83	0.23
b) Any NaM‐ASM	1.06	0.53–2.10	0.88
Active TCS	3.79	1.99–7.20	<0.001
Any cardiac history	1.74	0.54–5.60	0.35

Abbreviations: aHR = adjusted hazards ratio; CI = confidence interval; NaM‐ASM = sodium channel modulating antiseizure medication; TCS = tonic–clonic seizure.

## 3 DISCUSSION

This study found no significant difference in lamotrigine or NaM‐ASM prescription at the time of EMU admission between cases who subsequently died of SUDEP and controls, and no evidence for an increased risk of SUDEP with lamotrigine or NaM‐ASM prescription immediately following admission, and up to 16 years later. Notably, a history or current diagnosis of structural heart disease at the time of EMU admission was not a risk factor for SUDEP, irrespective of lamotrigine or NaM‐ASM use. After controlling for confounding factors, those with TCSs in the preceding 12 months prior to EMU admission remained over two times more likely to die of SUDEP later in life than those without.

This study adds to a growing body of evidence that lamotrigine is unlikely to be associated with an increased risk for SUDEP, and supports previous research that achieving TCS control is the strongest modifiable factor for reducing SUDEP risk.^4^ Given complete ASM data at death was not available, these data are particularly relevant to the peri‐admission period of epilepsy patients taking lamotrigine or other NaM‐ASMs,

### 3.1 Strengths and limitations

The strengths of this study lie in its large population sampling across multiple EMUs, two countries, and health systems over a 20‐year period, and its successful linkage to national death registries to ensure accurate identification of decedents. SUDEP determination was independently made by three experienced practitioners using well‐established criterion and important clinical information from medical records at the time of admission were gathered, including the history of structural cardiac disease, seizure frequency, and clinical epilepsy characteristics. Unfortunately, as ASM data was not reliably available for matched controls at the time of follow‐up, the role of lamotrigine and NaM‐ASMs prescription at the time of death could not be directly assessed. Reassuringly, the proportions of SUDEP cases in each ASM group at EMU admission were comparable to those at the time of death, and few SUDEP cases were on different ASMs at the time of death to that at the time of admission. In addition, the conducted sensitivity analyses to assess the impact of misclassification of ASM use at death found the potential for these were unlikely to alter the results of our study. The role of long‐term ASM use and disease course over time were not also assessed in this study. Additionally, differences in the requirement for EMU‐captured seizures as inclusion criteria for epilepsy cases between Australian and US centers could confound results.

### 3.2 Implications

This study provides additional evidence that lamotrigine and other NaM‐ASMs are unlikely to be associated with an increased risk of SUDEP in the immediate years following use, up to 16 years. This study and other population‐based studies provide reassurance to clinicians prescribing lamotrigine and other NaM‐ASMs that an increased immediate and long‐term risk of SUDEP associated with these ASMs is likely to be low. Reducing seizure frequency, particularly TCSs, remains the strongest modifiable factor for lowering an individual's risk of SUDEP, and lamotrigine and other NaM‐ASMs remain important treatment options for achieving seizure control in many patients. As the FDA recommends caution in the use of these ASMs in patients with structural or ischaemic cardiac disease, population‐based studies examining all‐cause mortality in the general population with ASM use are also needed, although it was reassuring that this study found no association between a history of cardiac disease and SUDEP in patients taking lamotrigine or other NaM‐ASMs.

## AUTHOR CONTRIBUTIONS

**R. Nightscales** reports no disclosures relevant to the manuscript. **S. Barnard** reports no disclosures relevant to the manuscript. **J. Laze** reports no disclosures relevant to the manuscript. **G. Tao** reports no disclosures relevant to the manuscript. **C. Auvrez** reports no disclosures relevant to the manuscript. **S. Sivathamboo** is supported by a Bridging Postdoctoral Fellowship from Monash University (BPF20‐3253672466) and the Victorian Medical Research Acceleration Fund. She reports salary support paid to her institution from Kaoskey and Optalert for clinical trial‐related activities; she receives no personal income for these activities. **Z. Chen** is supported by an Early Career Fellowship from the National Health and Medical Research Council (GNT1156444). **W. D'Souza** receives salary support from The University of Melbourne. He has received travel, investigator‐initiated, scientific advisory board, and speaker honoraria from UCB Pharma Australia & Global; investigator‐initiated, scientific advisory board, travel, and speaker honoraria from Eisai Australia & Global; advisory board honoraria from Liva Nova; educational grants from Novartis Pharmaceuticals, Pfizer Pharmaceuticals, and Sanofi‐Synthelabo; educational; travel and fellowship grants from GSK Neurology Australia, and honoraria from SciGen Pharmaceuticals. He has shareholdings in the device company EpiMinder. **M.J. Cook** receives honoraria from UCB Pharma and Eisai. He is a shareholder and Chief Medical Officer for Epiminder Pty Ltd and Seer Medical Pty Ltd. **P. Kwan** is supported by a Medical Research Future Fund from the National Health and Medical Research Council of Australia (APP1136427) and the Victorian Medical Research Acceleration Fund. He reports grants and personal fees from Eisai, UCB Pharma, and LivaNova; reports grants from Zynerba, Biscayne, and GW Pharmaceuticals; and has received travel, speaker honoraria, or consultancy fees from Sun Pharmaceuticals, Supernus Pharmaceuticals, Novartis, and Eisai. **D. Friedman** receives salary support for consulting and clinical trial‐related activities performed on behalf of The Epilepsy Study Consortium, a non‐profit organization. Dr. Friedman receives no personal income for these activities. NYU receives a fixed amount from the Epilepsy Study Consortium toward his salary. Within the past 2 years, The Epilepsy Study Consortium received payments for research services performed by Dr. Friedman from: Alterity, Baergic, Biogen, BioXcell, Cerevel, Cerebral, Jannsen, Lundbeck, Neurocrine, SK Life Science, and Xenon. He has also served as a paid consultant for Neurelis Pharmaceuticals and Receptor Life Sciences. He has received travel support from the Epilepsy Foundation. He has received research support from NINDS, CDC, and Epitel unrelated to this study. He serves on the scientific advisory board for Receptor Life Sciences. He holds equity interests in Neuroview Technology. He received royalty income from Oxford University Press. **S.F. Berkovic** is supported by a Program Grant from the National Health and Medical Research Council of Australia (APP1091593). He reports grants from Eisai, UCB Pharma, and SciGen; has a patent for SCN1A licensed to various diagnostic companies with no financial return, a patent for PRRT2 gene licensed to Athena Diagnostics, and a patent for Diagnostic and Therapeutic Methods for Epilepsy and Mental Retardation Limited to Females (EFMR) licensed to Athena Diagnostics. **P. Perucca** is supported by an Early Career Fellowship from the National Health and Medical Research Council (APP1163708), the Epilepsy Foundation, The University of Melbourne, Monash University, Brain Australia, and the Weary Dunlop Medical Research Foundation. He has received speaker honoraria or consultancy fees to his institution from Chiesi, Eisai, the limbic, LivaNova, Novartis, Sun Pharma, Supernus, and UCB Pharma. He is an Associate Editor for Epilepsia Open. **O. Devinsky** is supported by Finding a Cure for Epilepsy and Seizures (FACES), the National Institute of Neurological Disorders and Stroke (NINDS), the National Institute of Mental Health (NIMH), Multidisciplinary University Research Initiatives (MURI), Centers for Disease Control and Prevention (CDC) and National Science Foundation (NSF). O. Devinsky has equity interests in Qstate Biosciences, Tevard Biosciences, Regel Therapeutics and Script Biosciences, Tilray, Receptor Life Sciences, Empatica, Engage, Egg Rock/Papa & Barkley, Rettco, SilverSpike, and California Cannabis Enterprises (CCE). He is an investigator for PTC Therapeutics, Inc., Stoke Therapeutics, Marinus, Ovid, and GW Pharmaceuticals. He has consulted for Xenon, Zogenix, Marinus, and BridgeBio. **T.J. O'Brien** is supported by a Program Grant (APP1091593) and Investigator Grant (APP1176426) from the National Health and Medical Research Council of Australia and the Victorian Medical Research Acceleration Fund. He reports grants and consulting fees to his institution from Eisai, UCB Pharma, Praxis, Biogen, ES Therapeutics, and Zynerba.

## FUNDING INFORMATION

Finding A Cure for Epilepsy and Seizures, and the Australian National Health and Medical Research Council.

## ETHICS STATEMENT

Australian Human Research Ethics Committee approval for the study was provided by St Vincent's Hospital, Melbourne Health (The Royal Melbourne Hospital), and Austin Health (The Austin Hospital). Approval for data linkage to the Australian National Death Index was granted by the Australian Institute of Health and Welfare (AIHW) Ethics committee. Access to the Australian National Coronial Information System was granted by the Justice Human Research Ethics Committee. Approval for inclusion of US cases was granted by the NYU Institutional Review Board. We confirm that we have read the Journal's position on issues involved in ethical publication and affirm that this report is consistent with those guidelines.

## Supporting information


Appendix S1

## Data Availability

Data used in the analysis are available from the corresponding author upon request from any qualified researcher for a period of 12 months following publication.
