# Supplementary material for: Risk of sudden unexpected death in epilepsy (SUDEP) with lamotrigine and other sodium channel‐modulating antiseizure medications
Source: Epilepsia Open. 2023 Feb 15;8(2):334–45. doi: 10.1002/epi4.12693 (PMC10235563; doi:10.1002/epi4.12693)
Supplement: Supplementary file 1 — Appendix S1 [file EPI4-8-334-s001.docx]

**Appendix 1: Author contributions**

| Name | Location | Contribution |
| --- | --- | --- |
| Russell Nightscales, MD | The Alfred Hospital, Melbourne, Australia | Drafted and revised the manuscript for intellectual content, major role in acquisition of data, and contributed to the interpretation of data |
| Sarah Barnard, MD MIPH | Monash University, Melbourne, Australia | Drafted and revised the manuscript for intellectual content, major role in acquisition of data, and contributed to the interpretation of data |
| Juliana Laze, MD | New York University Grossman School of Medicine, New York, USA | Major role in acquisition of data, contributed to the interpretation of data, and revised the manuscript for intellectual content |
| Zhibin Chen, PhD | Monash University, Melbourne, Australia | Performed statistical analyses, revised the manuscript for intellectual content, and contributed to the interpretation of data |
| Gerard Tao, MD | The Royal Melbourne Hospital, Melbourne, Australia | Contributed to the acquisition of data, and revised the manuscript for intellectual content |
| Clarissa Auvrez, MD | Melbourne Health, Melbourne, Australia | Contributed to the acquisition of data, and revised the manuscript for intellectual content |
| Shobi Sivathamboo, PhD | Monash University, Melbourne, Australia | Critical revision of the manuscript for intellectual content. |
| Mark J. Cook, MD | St. Vincent’s Hospital, Melbourne, Australia | Critical revision of the manuscript for intellectual content. |
| Patrick Kwan, MD, PhD | Monash University, Melbourne, Australia | Critical revision of the manuscript for intellectual content. |
| Daniel Friedman, MD | New York University Grossman School of Medicine, New York, USA | Provided scientific direction and revised the manuscript for intellectual content. |
| Samuel F. Berkovic, MD | Austin Health, Melbourne, Australia | Principal investigator, provided scientific direction, reviewed clinical data, contributed to the interpretation of data, and critical revision of the manuscript for intellectual content. |
| Wendyl D’Souza, MDChB, PhD | St. Vincent’s Hospital, Melbourne, Australia | Principal investigator, provided scientific direction, reviewed clinical data, contributed to the interpretation of data, and critical revision of the manuscript for intellectual content. |
| Piero Perucca, MD, PhD | Monash University, Melbourne, Australia | Provided scientific direction, reviewed clinical data, contributed to the interpretation of data, and critical revision of the manuscript for intellectual content. |
| Orrin Devinsky, MD | New York University Grossman School of Medicine, New York, USA | Principal investigator, provided scientific direction, and critical revision of the manuscript for intellectual content. |
| Terence J. O’Brien, MD | Monash University, Melbourne, Australia | Principal investigator, designed and conceptualized the study, provided scientific direction, reviewed clinical data, contributed to the interpretation of data, and critical revision of the manuscript for intellectual content. |
